# A multi-site randomized study to compare the effects of Eye Movement Desensitization and Reprocessing (EMDR) added to TAU versus TAU to reduce craving and drinking behavior in alcohol dependent outpatients: study protocol

**DOI:** 10.1186/s12888-015-0431-z

**Published:** 2015-03-18

**Authors:** Wiebren Markus, Gerdien H de Weert – van Oene, Eni S Becker, Cor AJ DeJong

**Affiliations:** 1 IrisZorg, Institute for Addiction Care, Sheltered Housing and Social Support Services, Kronenburgsingel 545, P.O. box 351, 6800 AJ Arnhem, Netherlands; 2 Nijmegen Institute for Scientist-Practitioners in Addiction (NISPA), Radboud Universiteit Nijmegen/ACSW, P.O. box 9104, 6500 HE Nijmegen, Netherlands; 3 Behavioral Science Institute (BSI), Radboud University Nijmegen, P.O. box 9104, 6500 HE Nijmegen, The Netherlands; 4 Victas, Centre for Addiction Treatment, Tolsteegsingel 2A, 3582 AC Utrecht, The Netherlands

**Keywords:** Eye movement desensitization and reprocessing, EMDR, Alcohol, Craving, Addiction, Working memory, Randomized controlled trial, Study protocol

## Abstract

**Background:**

Addiction constitutes a major public health problem, and despite treatment, relapse rates remain very high. Preliminary findings suggest that Eye Movement Desensitization and Reprocessing (EMDR), an evidence-based treatment for PTSD, may also reduce craving and relapse rates when applied in substance abuse. This study aims to determine the feasibility, efficacy and effectiveness of EMDR when added to treatment as usual (TAU) for addiction in alcohol dependent outpatients, compared to TAU only.

**Methods/Design:**

A single blinded study in which 100 adult patients with a primary DSM-IV-TR diagnosis of alcohol dependence or abuse receiving treatment in one of six Dutch outpatient addiction care facility sites, will be enrolled. After baseline assessment participants will be allocated to one of two treatment conditions (allocation ratio of 1:1) using a stratified (per site, per care pathway), blocked randomization procedure. The intervention consists of EMDR (seven weekly 90 minute sessions) + TAU or TAU only. Assessments are scheduled pre-treatment (t0), post-treatment (t0 + eight weeks), and one and six months post treatment. The effects of both treatment arms are compared on indices of (a) drinking behavior, (b) mediators, moderators and predictors of treatment outcome, (c) quality of life and d) safety, acceptability and feasibility of treatment.

Repeated measures ANOVA’s will be conducted using an intention-to-treat and per-protocol approach. Multiple imputation will be used to deal with missing values when possible.

**Discussion:**

This study adapts and extends the standard EMDR treatment for traumatized patients for use with patients with alcohol use disorders without psychological trauma.

**Trial registration:**

ClinicalTrial.gov: NCT01828866

## Background

In 2013 in the Netherlands, 46% of all treatment seeking patients with an addiction problem (more than 65.000) sought help for alcohol-related problems [1]. However, despite treatment, addictions such as alcohol dependence are characterized by high relapse rates [2]. For 1-year outcomes across alcohol, nicotine, and illicit drug abuse, more than 85% of patients relapse within 1 year of treatment.

Many factors are implicated in the genesis, escalation and maintenance of addiction. Here some known predictors of alcohol use are discussed before we introduce a relatively new approach to target some of these mechanisms in order to reduce drinking behavior. A multi-factorial approach is adopted to gain insight into possible mechanisms of change regarding this approach.

### Mediators, moderators and predictors of alcohol use and relapse

Addiction can be conceptualized as a cyclic process of anticipation, intoxication, and withdrawal [3]. The anticipation stage is considered a key element of relapse and often takes the form of intense craving. Craving refers to a compelling or intense urge, desire or intention to ingest a drug [4]. High baseline levels of alcohol craving may define a subtype of alcohol dependence that is less responsive to treatment and predict the chance of relapse after discharge [5]. In addition to craving itself it may be interesting to look at related concepts, such as desire thinking and rumination, which seem to augment craving.

Desire thinking refers to the elaboration of cognitions and imagery in working memory which may escalate craving intensity [6]. It correlates with escalating levels of drinking status [7]. Desire thinking is regarded as a specific type of perseverative thinking, such as rumination or worry [6]. Interestingly, rumination predicts long term outcomes of addiction treatment in alcohol-dependent patients [8]. Rumination increases craving in alcohol-dependent drinkers but not in problem and social drinkers [9]. So both desire thinking and rumination increase or intensify craving. Since rumination results in increased negative affect [10], it is no surprise that both low positive and high negative affect are associated with relapse in addiction [11]. So when levels of desire thinking and rumination are reduced, craving and negative affect may also be reduced, which would result in lower relapse rates.

In addition to these subjective processes, automatic implicit processes such as attentional biases have also been implicated in the maintenance of addiction [12,13]. Since alcohol attentional biases have a reciprocal excitatory relationship with craving, reductions in attentional bias should predict reductions in craving and drinking behavior. Whereas craving, desire thinking, rumination and negative affect are typically measured by subjective participant report, the strength of implicit processes may be measured more objectively in paradigms using changes in reaction times as primary outcome.

Other known predictors of alcohol treatment outcome which will be assessed in this study are baseline alcohol consumption, dependence severity, employment, gender, psychopathology rating, treatment history, motivation, socioeconomic status/income and self-efficacy [14]. Increased self-efficacy during treatment predicts a positive treatment outcome while an excess of self-efficacy can be inconsistent with the actual ability of a patient to resist use and may result in drop-out.

In sum, there is a large body of research on predictors of alcohol treatment outcome. These factors should be taken into account when aiming to reduce relapse rates since they can help identify at an early stage which interventions may have the highest clinical effectiveness over time. In addition, they provide important targets for direct intervention.

In this study we focus on an approach which targets episodic drug memories to reduce craving, alcohol consumption and relapse rates: Eye Movement Desensitization and Reprocessing (EMDR) [15].

### Research on EMDR in addiction

EMDR is a structured treatment for posttraumatic stress disorder (PTSD) [16]. The core of the procedure entails the identification and reactivation of specific memory representations in working memory while the patient follows the therapist’s horizontal hand movements with her or his eyes. Since this taxes working memory and working memory is limited in capacity, the eye movements compete for attention with the reactivated memory [17]. Every 30 seconds, the patient reports spontaneous associations, which form the focus of attention during a new set of eye movements. Over time the memory representation gets less vivid and emotionally charged (‘desensitisized’) while a new, more adaptive self-perspective tends to arise [18]. The EMDR procedure can also be used to desensitize dysfunctional positive memory representations [19] or that of anticipated events [20].

Research has clearly demonstrated EMDR’s effect in the treatment of PTSD [21]. Some have suggested that, on the basis of an emerging body of research, EMDR may be regarded as a general model for psychotherapy for a wider range of conditions, such as addiction [22]. However, research on the efficacy and effectiveness of EMDR on addiction is still limited, consisting mostly of anecdotal reports or case studies of substance-related [23-31] as well as behavioral addictions [32-37]. Although most of these reports and case studies found positive results, some found mixed [30] or negative results [24,25]. The interpretation of results is further complicated by the fact that different approaches were used [36,38,39].

To date, one RCT has been published [15]. Detoxified, in-patient alcohol dependent patients received TAU plus two one-hour sessions of EMDR targeting memory representations of intense craving or relapse. Controls received TAU only. Decreased craving at 1-month follow-up, as well as a lower relapse rate at 1- and 6-month follow-up were found in the experimental group. However, the sample size was small (n = 34), attrition was high, assessment depended fully on self-report and treatment was applied by the researcher. This raises questions about the actual efficacy and effectiveness of EMDR. In addition, although no adverse effects were reported, this study showed that relapse or study drop-out rates may still be substantial and should be taken into account when determining the feasibility of the treatment.

In sum, although limited data and methodological issues prohibit firm conclusions, further research on the efficacy of EMDR in addiction is warranted.

### Objectives

#### Primary objective – effects of treatment on drinking behavior

This study examines the efficacy and effectiveness of EMDR + TAU versus TAU only on drinking behaviour in a sample of alcohol dependent participants.

We operationalize drinking behavior as: a) changes in number of heavy drinking days (days on which ≥ 5 standard drinks of alcohol are consumed); b) time to first alcohol drink (only relevant if abstinence was reached before the previous assessment); c) changes in total drinks consumed in the past 30 days; and d) changes in average drinks per occasion in the past 30 days (total drinks divided by drinking days). Participant-reported information is backed-up with changes in relevant biomarker data (derived from blood samples at all time-points): carbohydrate-deficient transferrin (CDT) combined with serum γ-glutamyltransferase (GGT). An overview of the measurements at different time points is given in Table 1.

**Table 1:** **Measurements of primary, secondary and tertiary objectives: drinking behavior, mediators, moderators and predictors and quality of life**

	Outcome	Measurement	Baseline screening	T0	T1	T2	T3
		-interview (i)		Pretreatment	Posttreatment	1 month FU	6 months FU
		-self-report (s)					
		-reaction time (r)					
		-bloodsample (b)					
1	Recent and lifetime use, indications for psychiatric or medical evaluation, addiction treatment history and current abuse or dependence (DSM-IV)	MATE (part 1–4) (i)	X				
2	Comorbid psychiatry (DSM-IV)	MINI-plus (i)	X				
3	Motivation to change	RCQ-D (s)		X			
4	Harmful alc. use	AUDIT (30 d) (s)		X	X	X	X
5	Self-efficacy	SELD (s)		X	X	X	X
6	Health outcome	EQ-5D (s)		X	X	X	X
7	Quality of life	CRA-HS (s)		X	X	X	X
8	Alcohol craving	PACS (s)		X	X	X	X
9	Desire thinking	DTQ (s)		X	X	X	X
10	Pos. and neg. affect	PANAS-SF (s)		X	X	X	X
11	Rumination	PTQ (s)		X	X	X	X
12	Drinking behavior	TLFB (30 d) (i)		X	X	X	X
13	Alcohol attentional bias	Alcohol Stroop (r)		X	X	X	X
14	Alcohol implicit assoc.	Valence IAT (r)		X	X	X	X
15	Biomarkers chron. alc. use	GGT + CDT (b)		X	X	X	X

*AUDIT* Alcohol Use Disorders Identification Test, *CDT* carbohydrate-deficient transferrin, *CRA-HS* Community Reinforcement Approach Happiness Scale, *DSM-IV* Diagnostic and Statistical Manual – 4th ed., *DTQ* Desire Thinking Questionnaire, *EQ-5D™* EuroQol - 5 dimensions, *GGT* serum γ-glutamyltransferase, *IAT* Implicit Association Task, *M.I.N.I.-plus* M.I.N.I. International Neuropsychiatric Interview-Plus, *PACS* Penn Alcohol Craving Scale, *PANAS-SF* Positive And Negative Affect Schedule – Short Form, *PTQ* Perseverative Thinking Questionnaire, *RCQ-D* Readiness to Change Questionnaire - Dutch version, *SELD* Self-Efficacy List for Drug users, *TLFB* Timeline Followback.

The primary outcome variable is changes in number of heavy drinking days since escalating numbers of heavy drinking days may best reflect a (temporarily) loss of control of drinking behavior, while the other behavioral outcomes could also reflect (to some degree) social or controlled drinking behavior.

#### Secondary objectives – mediators, moderators and predictors of treatment outcome

To explore mechanisms of change, variables that are potential predictors, mediators or moderators of treatment outcome will be examined (see Table 1). We distinguish:

1. Dependent variables:
  - Psychological (participant report): desire thinking, craving, rumination, negative affect and coping self-efficacy,
  - Psychological (reaction times): alcohol attentional bias and implicit associations towards alcohol cues.
2. Covariates:
  - Measured at baseline: demographic characteristics, time-in-treatment, comorbid psychopathology, alcohol consumption, alcohol dependence severity, other substance use and readiness to change;
  - Measured between baseline and follow-up: completer status, time-in-treatment, treatment intensity, the use of psychopharmaca, anticraving (naltrexone and acamprosate) or alcohol abstinence enforcing medication (disulfuram).

#### Tertiary objective – effects of treatment on quality of life

The aim of addiction treatment is recovery which can be defined as abstinence plus improved quality of life [40]. Therefore, quality of life will be assessed in addition to the drinking related variables mentioned above (see Table 1).

#### Quarteniary objectives – safety, acceptability and feasibility of treatment

Expectancies and experiences about treatment of both participants and therapists may affect treatment outcome. Therefore harm expectancy and experience, expected and experienced burden, credibility of treatment and treatment adherence will be assessed.

## Methods/design

### Design

This study is a randomized controlled trial with two arms: EMDR + TAU versus TAU. The two groups are compared pre-treatment (T0), post treatment (T1 = T0 + 8 weeks) and at follow up one (T2) and six months (T3) post treatment, on variables linked to the research questions. This amounts to a 2 × 4 design with the between factor condition (EMDR + TAU vs. TAU) and within factor time (pre- x post intervention × 1 × 6 months follow-up).

Both groups will be assessed at the same time-points, whereby a substantial part of both groups may still receive TAU at follow-up after 1 month, but may no longer receive TAU at follow-up after 6 months.

The current study is hybrid in that it tries to broaden the generalizability of the outcomes by recruiting a representative, but complex sample while maintaining some of the rigor of efficacy research (e.g. using training and supervision, monitoring of and feedback on treatment fidelity). This allows to analyze data from an effectiveness (outcome in real life circumstances, using all data, including imputed data in case of study drop-out etc.) and an efficacy (outcome under ideal circumstances using only completers data) viewpoint.

The design of this study was approved by the Medical Ethics Committee of Medisch Spectrum Twente and is registered as study NL43892.044.13.

### Participants and recruitment

**Figure 1:**
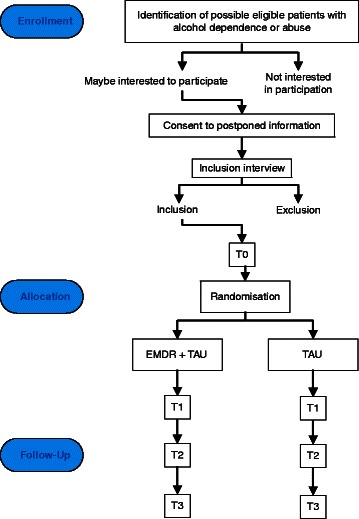
**Flow Diagram RCT.**

Adult patients, 18 years or older, with a primary DSM-IV-TR [41] diagnosis of alcohol dependence or abuse are recruited from six outpatient sites of IrisZorg, an addiction treatment organization in the Netherlands. The trial process starts with a global identification of possible eligible patients (on the basis of known in- and exclusion criteria, see next paragraph) by a triagist when they seek treatment. If they agree to be informed about the study, they are contacted by a research assistant (RA1, a psychologist) to provide additional information and to answer questions. Participants consent to postponed information after they receive limited oral and written information to be able to blind them as much as possible from later group allocation using the procedure outlined by [42]. After consent, an appointment is made for an inclusion interview. If included, participants undergo a pre-treatment assessment (T0) (see Figure 1).

### Inclusion and exclusion

The inclusion criteria are:

1. Age 18 or older;
2. Fluent in Dutch speaking and reading abilities (clinical observation by RA1) and;
3. A primary DSM-IV-TR diagnosis of alcohol dependence or abuse.

This last criterion is assessed during the regular intake procedure by a health care or clinical psychologist.

Exclusion criteria are:

1. Meeting the DSM-IV-TR criteria for current PTSD;
2. Meeting the DSM-IV-TR criteria for current dependence and regular (at least once per week in the last 2 weeks before eligibility screening) use of substances other than alcohol or nicotine;
3. Current (in the last two weeks before eligibility screening) regular alcohol use (at least > 21E (women) or > 28E (men) per week) and;
4. Severe, current (since the start of regular treatment) psychiatric symptoms (especially manic, psychotic, suicidal and aggressive symptoms) that interfere with TAU.

These criteria are assessed during intake by a health care or clinical psychologist and/or (re)assessed by RA1 during screening. RA1 uses segments of the Mini-International Neuropsychiatric Interview-Plus (MINI-Plus [43]), the Measurements in the Addictions for Triage and Evaluation (MATE 2.1 [44]) and/or clinical observation as well as information from the patients dossier.

When RA1 detects psychopathology not detected during regular intake, the participant is informed as well as his/her regular therapist to ensure further diagnostics and treatment is provided, if necessary.

### Randomization and allocation

After inclusion, participants are randomized to either EMDR + TAU or “TAU only” by another RA (RA2, a psychologist), not involved in the assessments. Participants are allocated to their treatment condition based on a randomization code. RA2 generates a randomization sequence before the recruitment phase starts using freely available software [45].

The randomization sequence is stratified by participating facility (a total of six sites), further subdivided by a maximum of three care pathway (‘youth’ (youngsters up to 23 years old), ‘addiction’ (adult patients with primarily addiction) or ‘addition, care and housing’ (adult patients with problems on multiple life domains in addition to addiction and often severe psychiatric problems), if there is a participating EMDR therapist available in that striatum. EMDR therapists may work at multiple facilities and/or care pathways and thus see participants from different strata. This amounts to a stratified randomization with a 1:1 group allocation within each stratum, using block sizes of 4.

When participants are allocated to TAU only, no action is taken (since RA1 already planned subsequent assessments, independent of allocation). When they are allocated to EMDR + TAU, RA2 informs participants by email about EMDR with additional written information. RA2 also informs a participating EMDR therapist of the same facility where the participant receives TAU. The EMDR therapist contacts the participant and provides additional information about EMDR to the participant and plans seven weekly EMDR sessions.

Great care is taken so that all those involved in the study are blinded as much as possible. Participants, RA1 (the assessor) and the principal investigator are blinded to group allocation while EMDR therapists are blinded to outcome of assessments. In addition, the principal investigator was not involved in the treatment of participants.

### Power and sample size calculation

Since two active treatment arms are compared and the sample is expected to be relatively heterogeneous regarding baseline variables such as dependence severity, drinking status and other substance use, it is estimated that the effect size will be relatively small. Clinical relevant differences on the primary outcome variable (changes in number of heavy drinking days in the previous 30 days) were calculated on the basis of data of a recent study [46]. In patients with an alcohol-use disorder referred to in- or outpatient alcohol treatment the mean baseline score for heavy drinking days (≥5 drinks in a single day, over the past 30 days) was 16.33 (SD 11.99). Treatment outcome was such that undertreated patients (<75% attendance of the number of sessions recommended by treatment allocation guidelines, n = 618) showed a reduction of 8.7 heavy drinking days (±12.5) while overtreated patients (>110% attendance of sessions recommended) showed a reduction of 12.5 heavy drinking days (±12.2, n = 362). The difference between under- and overtreated patients is at least 3.8 heavy drinking days over the past 30 days which corresponds to an effect size of *d* = 0.31 or *f* = 0.15).

Subsequent poweranalysis in G*Power version 3.1.2. [47] (ANOVA: repeated measures, within-between interaction) indicated a sample size of 74 to demonstrate this effect (keeping α at 0.05 and power at 0.80 and using 2 groups and 3 measurements (concerning changes in the number of heavy drinking days between 4 time points)). Attrition is estimated to be relatively high at 33%. Therefore we add 26 participants (100 patients in total, 50 in each treatment arm), to meet the required sample size.

To minimize missing values RA1 will use email, text messages, telephone calls, and/or contacts with participants’ regular therapists to remind participants about their assessment appointments. All participants will receive financial compensation of 25 euros (in the form of a gift cheque) for every complete assessment at T2 and T3, regardless of whether or not the participant drops out of treatment.

### Interventions

#### Treatment as usual (TAU)

All participants already receive TAU at the moment they are enrolled and will continue to receive TAU for as long as indicated by their regular therapists but at least until T1. TAU may differ in intensity or additional components delivered (e.g. family treatment) between participants. In IrisZorg, most out-patients of care pathway ‘youth’ and ‘addiction’ receive (Adolescent) Community Reinforcement Approach ((A)CRA) treatment, consisting of several interventions, based on behavioral therapy principles [48]. Elements may include: functional analysis, training communication and/or problem-solving skills, sobriety sampling, social networking, learning to refuse substances, increasing alternative reinforcing activities, relapse management and medication monitoring. Treatment is individual but may also be partly groupwise. Patients receive TAU on a weekly basis by a health care worker.

Alternatively, some patients in care pathway ‘young’ may receive an intensive family treatment for youngsters up to 20 years old (and their families), who still live at home (or intend to go back to live at home again) and where multiple life domains may be affected (e.g. family, school, mental health, criminal behavior etc.): Multidimensional Family Therapy (MDFT [49]). Patients of care pathway ‘addition, care and housing’ receive Function Assertive Community Treatment (FACT [50]), a multidisciplinary, outreaching approach aimed at stabilization, treatment and recovery.

TAU is supervised by a clinical psychologist or psychiatrist. If necessary, treatment may be supplemented with medical care (such as, but not limited to medication), individual psychological care or relationship or family therapy. Incidentally or periodically, additional in-patient treatment may be necessary. CRA and MDFT typically lasts 6 months (in some cases CRA may be limited to 3 or extended to 9 months) while FACT may endure much longer.

‘Time-in-treatment’ before intervention and during the course of the study as well as main components and treatment intensity of TAU will be registered. The use of psychofarmaca, craving reducing (acamprosate, naltrexone) or abstinence enforcing medication (disulfuram) will be monitored.

### Eye movement desensitization and reprocessing (EMDR)

Participants allocated to the EMDR + TAU group will receive a manualized intervention. This set of EMDR protocols was developed by the principal investigator and the EMDR International Association (EMDRIA)-approved consultant involved in this study as a trainer. It incorporates elements of existing EMDR addiction protocols [35,37,38], related work [51] and insights on positive and prospective memory representations [19,20]. Based on pilot treatments, the intervention manual was fine-tuned before the recruitment phase.

Participants receive a maximum of seven weekly, 90-minute EMDR sessions provided by health care psychologists (who at least completed an EMDRIA-approved Basic Training program). The EMDR therapists will be trained in the use of the intervention manual and will be supervised monthly by the EMDR consultant. All EMDR sessions are video-recorded for fidelity rating and supervision.

Session one starts with instruction on some rules and the rationale for EMDR and its use in addiction. Then the treatment goal (controlled drinking or abstinence) is established. Subsequently participants are instructed to imagine their positive treatment goals vividly. After that both negative associations with long-term abstinence (if present) and positive memories associated with alcohol use are targeted with EMDR. At the end a homework diary is presented and participants are instructed to keep it daily, between sessions. It helps to register daily craving, alcohol use, possible adverse and/or positive events and help participants to prepare for the next session by identifying possible targets. Subsequent sessions focus on memory representations associated with loss of control, self-efficacy undermining beliefs, trigger situations, and anticipated relapse. Finally the focus shifts to feeling more empowered in trigger situations. Therapists are instructed to keep the duration of sessions as constant as possible, selecting only the most relevant targets. In some cases, fewer sessions may be required and provided.

### Termination of treatment

Regarding EMDR treatment, completers are defined as completing five or more of the scheduled seven EMDR sessions, allowing for some flexibility on the basis of clinical need. Those who attend four EMDR sessions or fewer for any reason comprise a premature termination of treatment (PTT) group.

Regarding TAU, a similar distinction is made whereby a completer attended five or more weeks of treatment (at least one face-to-face contact weekly) between T0 and T1 while those who attend four weeks or less of TAU fall in the PTT category.

### Data collection

Subjects participate in the study for 8 months (from T0 to T3). Patients in both treatment conditions will be assessed identically (see Table 1). Two instruments are only used during the inclusion interview: the MINI-Plus [43] and the MATE 2.1 [44]. At T0, RA1 (blinded to treatment allocation) assesses baseline measurements of all primary, secondary, tertiary and quaternary variables. At T1 (post treatment), T2 (follow-up after 1 month) and T3 (follow-up after 6 months) all but one (the ‘readiness for change’ as measured with the RCQ-D [52] is only assessed at baseline) measurements are repeated by RA1.

For participants in the EMDR + TAU group additional assessments take place within (pre and post levels of distress, craving or positive affect of memory representations on a 11-pt Likert scale) and between (daily diary: number of drinks, intensity and frequency of craving, adverse events) sessions. These assessments are an integral part of the EMDR intervention manual and can therefore only be used to explore changes during the course of EMDR within this group. EMDR therapists also fill in a questionnaire after the last EMDR session of each participant regarding perceived and experienced treatment burden credibility and harm, before, during and after treatment.

RA1 also gathers demographic data: age, sex, social-economic and marital status, educational level, and ethnicity. Participant-report questionnaires (no 3–11 in Table 1 below [6,52-59]) will be provided digitally using Perseus web survey software [60]. The alcohol Timeline Followback interview (TLFB; [61]) is conducted face-to-face by RA1.

The alcohol Stroop [62] and valence IAT [63] will be administered using the program Inquisit 3 [64] for computerized assessment.

After each on-site assessment, participants are referred to a laboratory nearby for collection of blood samples to determine GGT and CDT [65]. Results are interpreted by an independent physician from IrisZorg. The results will be discussed with the participants after the last assessment by this physician, if relevant. In the case that lab results merit intervention before the last follow up assessment, the physician will notify the regular addiction care physician or general practitioner as soon as possible.

### Instruments

#### Measurements - inclusion

##### Measurements in the addictions for triage and evaluation (MATE 2.1 [44])

The MATE is composed of 10 modules made up of a set of existing psychometrically validated short instruments from the public domain. In this study, RA1 only uses parts 1–4 to screen for eligibility criteria.

- Module 1: ‘USE’, is an interview used to determine the severity of the use of psychoactive substances in the previous 30 days as well as life-time. Inventarisation of substances (including nicotine and gambling), the level of use and typical form or route of application are reported. Use (in standard units) in the past period and use on a typical day of use are reported, as well as the number of years of regular use. The primary problem substance or behaviour is identified.
- Module 2: ‘INDICATIONS PSYCHIATRIC OR MEDICAL CONSULT’. Medical themes interviewed: 1) use of medication for addiction; 2) use of medication for other somatic complaints; 3) symptoms that may be the result of severe physical health problems; 4) intoxication or (severe) withdrawal symptoms; and 5) pregnancy. Psychological/psychiatrical themes interviewed: 1) current and recent psychiatric or psychological treatment; 2) use of medication for psychiatric reasons; and 3) suicide risk and psychotic symptoms.
- Module 3: ‘ADDICTION TREATMENT HISTORY’. This part of the interview questions whether and if so how much previous addiction treatments the participant has undergone in the past 5 years.
- Module 4: ‘DEPENDENCE AND ABUSE’. The questions are derived from the section Alcohol & Drugs from the CIDI (Composite International Diagnostic Interview) 2.1 [66] in accordance with the diagnostic criteria for substance abuse or dependence of the DSM-IV-TR [41]. In this study, the questions will relate to alcohol use since this will be the primary problem substance.

##### Mini-international neuropsychiatric interview (MINI-plus [43,67])

This brief, structured interview allows for the coding of more than 60 variables, including DSM-IV-TR disorders (23 axis 1 disorders) and suicide risk at the time of the interview or at some time in the past. Psychometric evaluation of the English version of the MINI (a less detailed version of the MINI-plus) showed that inter-rater reliability is good while test-retest reliability is satisfactorily, expect for current manic episode. Most scales possess a satisfactory degree of predictive power but positive predictive power for lifetime bulimia nervosa, social phobia and generalized anxiety disorder is relative weak [68]. Content validity of simple phobia and generalized anxiety disorder, when compared to the CIDI [66], was also low.

The MINI-Plus employs different time frames for various disorders: current, past, or lifetime. For screening purposes, we will only focus on the ‘current’ timeframe.

#### Measurements – effects on drinking behavior

##### Alcohol use disorders identification test (AUDIT; [53,69]

The AUDIT is a 10-item, self-report measure assessing harmful alcohol consumption (items 1 to 3), drinking behavior (items 4 to 6), adverse reactions to alcohol (items 7 to 8), and alcohol-related problems (9 to 10). Scores on items 1 to 8 can range from 0 to 4, while items 9 and 10 are scored 0, 2, or 4. Higher scores indicate higher risk and range from 0 to 40. Scores between 8 and 12 indicate hazardous or harmful alcohol use, while scores 13 to 40 indicate likely alcohol dependence [70,71]. A high reliability, sensitivity, and specificity of the scale has been reported [53].

The timeframe normally used is that of the previous year. However, since we want to repeat the AUDIT assessment, this time frame would cause overlap between assessments. Therefore we will use a time-frame of 1 month.

##### Alcohol timeline followback (TLFB [60])

The Alcohol TLFB is a drinking assessment method that obtains estimates of daily drinking and has been evaluated with clinical and non-clinical populations. Using a calendar, people provide retrospective estimates of their daily drinking over a specified time period that can vary up to 12 months from the interview date. In this study retrospective estimates of the previous month will be used. Several memory aids can be used to enhance recall (e.g., calendar; key dates serve as anchors for reporting drinking; standard drink conversion). The Alcohol TLFB has been shown to have good psychometric characteristics with a variety of drinker groups, and can generate variables that provide a wide range of information about an individual’s drinking (e.g., pattern, variability, and magnitude of drinking). The method is recommended for use when relatively precise estimates of drinking are necessary, especially when a complete picture of drinking days (i.e., high- and low-risk days) is needed (evaluating drinking pre-posttreatment). Although Timeline summary data have been found to be generally reliable, as with all drinking assessment methods, exact day-by-day precision cannot be assumed or necessarily expected. Overall, the Alcohol TLFB method provides a relatively accurate portrayal of drinking, and has both clinical and research utility.

##### Serum γ-glutamyltransferase (GGT) and carbohydrate-deficient transferrin (CDT)

CDT is currently the most specific marker of alcohol abuse, and when combined with GGT using an equation [0.8*ln(GGT) + 1.3*ln(CDT)] a high sensitivity is reached without loss of specificity [64]. The Dutch multidisciplinary protocol for alcohol problems [72] also states that assessment of CDT levels, which may be combined with those of GGT, is suitable to monitor reduction of or abstinence from (chronic) alcohol use;

#### Measurements – mediators, moderators and predictors of treatment outcome

##### Readiness to change questionnaire (RCQ; [52,73]

The RCQ is an instrument which tries to capture the stage of change the patient is in, according to the stages of change model [74]. The RCQ has 12 items which have to be answered on a 5-point scale. There are three subscales reflecting pre-contemplation, contemplation and action. The instrument has a satisfactory internal consistency and, in most studies, a satisfactory test-retest reliability ([73], p. 752). The predictive validity of the original instrument is sufficient: the RCQ predicts changes in drinking behavior [75]. However, one study found that there was little agreement between self- and interviewer rating of the alcohol version of the RCQ and between self-report on different instruments relating to the same concepts [76].

The psychometric quality of the RCQ-D (translated by [73]) seems comparable to the original. Some negative formulated questions were transformed into positive formulations. Although the underlying transtheoretical model has been criticized [77], the questions in itself can provide insight into the motivation for change and drinking related appraisals at the moment of assessment. The instrument can assist in distinguishing those patients who are ready to change their drinking behavior and those who are probably in need of further motivational counseling [73].

##### Penn alcohol craving scale (PACS; [57])

The PACS is a measure of weekly craving. The PACS was selected because prior research has demonstrated the PACS to have greater predictive value for treatment outcomes compared to the Obsessive–Compulsive Drinking Scale or the Alcohol Urge Questionnaire [78]. The PACS consists of five items each scored 0–6 in increasing severity of craving.

The PACS has excellent internal consistency [57]. Predictive, construct, discriminant validity was demonstrated. The PACS is a reliable and valid measure of alcohol craving and can predict which individuals are at risk for subsequent relapse. Although the instrument has not been translated and validated in the Netherlands, we believe the brevity and psychometric qualities of the original instrument makes it suitable for the current study. A Dutch version (translated by two independent researchers and discussed after which consensus was reached on a final translation) was used here.

##### Desire thinking questionnaire (DTQ; [6])

The DTQ is based on the elaborated intrusion theory of desire [79]. Items are scored on a 4-point Likert-type scale (“Almost never”, “Sometimes”, “Often” or “Almost always”). Although the DTQ uses no timeframe, here we focus on the previous month in order to make sure there is no overlap in timeframe between subsequent assessments. Differentiated in a total score (10 items × 1–4 points (range 10–40 points)) and subscores for the factors Verbal Perseveration and Imaginal Prefiguration (5 items × 1–4 points (range 5–20 points)). The first factor relates to ‘repetitive self-talk regarding the need to achieve the desired target and self-motivated statements’. The latter relates to the ‘construction of mental images of the desired target or of its context of consumption.

Psychometric evaluation demonstrated an acceptable level of internal consistency [6]. A moderate correlation between desire thinking, craving and rumination indicated that these concepts can be considered as divergent constructs. Test-retest reliability for both factors (.66 and .56) and total score (.59) was acceptable.

Predictive validity was determined in a sample of alcohol abusers seeking treatment from a variety of alcohol services (n = 78). Both DTQ factors correlated positively and significantly with craving, but only ‘Verbal Perseveration’ correlated positively and significantly with the level of alcohol use (but did not predict level of alcohol use in addition to the variance explained by craving). DTQ factors predicted craving independently of level of alcohol use.

##### Positive and negative affect schedule – short form (PANAS-SF; [58]

This 10-item instrument is a translated, Dutch version of the International PANAS-SF (I-PANAS-SF; translated from English by comparing it to the validated 20-item Dutch version of the PANAS [80], itself a translation of the original English version of the PANAS [81]).

Each item refers to a mood state and participants rate the extent to which each mood state describes how they feel at the moment of testing on a scale ranging from 1 (never) to 5 (always). It captures two dimensions of mood: positive and negative affect. Low positive affect (PA) corresponds to lethargy and sadness, whereas high PA reflects high energy, concentration and pleasurable mood states. Negative affect (NA) refers to distress and unpleasurable mood states, with low NA reflecting a state of calmness and serenity. One study demonstrated adequate reliability of the PA and NA subscales of the I-PANAS-SF [81]. The instrument compares well with the full 20-item original of both correlating with the original full form and temporal stability. Although the validity of the instrument in relation to stress and psychopathology needs to be determined, the brevity and straightforwardness of the instrument make it useful for repeated measurements, especially in the multiple baseline study. In addition, both PA and NA seem to play a role in conferring risk and maintaining substance use [11]. Most short affect measures only capture aspects of NA.

##### Perseverative thinking questionnaire (PTQ; [59]

The 15-item PTQ was recently developed to provide a content-independent measure of repetitive negative thinking (RNT). Preliminary validation supported lower order factors representing 1) the core characteristics of RNT (repetitiveness, intrusiveness and difficulties with disengagement), 2) perceived unproductiveness of RNT and 3) RNT capturing mental capacity. The PTQ shows good reliability and validity and recently a Dutch version was validated [82]. Each item is rated on a 5-point scale ranging from 0 (never) to 4 (almost always). No timeframe is used.

##### Alcohol stroop (derived from classic Stroop test [62,83])

A modified, computerized Stroop color-naming task with alcohol-related words (alcoholic drinks) as well as neutral (non-alcoholic drinks) words is used. The stimulus words are presented on a 17 inch laptop (using Inquisit 3 desktop software) in 4 different colors (yellow, red, green and blue). Participants view 3 screens (each representing a matrix of 5 columns and 8 rows, equaling a set of 40 stimuli) with different stimulus sets in a randomized, counterbalanced (between subjects) fashion: a) 4 × 10 alcohol-related words (beer, wodka, wine, rum, port, cognac, whiskey, gin, liqueur and jenever), b) 4 × 10 neutral words (7-up, orange juice, apple juice, cola, Fanta, cassis, water, lemonade, spa, Pepsi) or c) 40 non-words (series of colored XXX). This amounts to 1 trial per set. Each of the ten selected words is presented four times per set in a fixed order. The order of the colors is fixed as well. The same word or ink color does not occur more than twice in a row. All stimuli appear in lowercase Arial font (regular). The font size is 14. The projected stimuli appeared on the computer screen as color words presented against a black background.

This task allows assessment of interference (on attentional processing) due to alcohol cues. Shorter response latencies on alcohol-related rather than neutral words are assumed to indicate a stronger alcohol-related bias. In the version used here, participants are required to respond vocally to stimuli. Participants only name the ink color, reading as fast as possible from left to right and from top to bottom. Timing was done manually: directly after the last ink-color is named, the experimenter clicks the mouse, recording the total time per set. Number of errors are recorded manually by the experimenter. Both the alcohol-related and neutral scores are corrected by subtracting the non-word score.

##### Implicit association task (IAT; [84]

Here the valence version [63] is used (derived from the Inquisit task library [85]). This IAT contains two sets of two word categories. The target words consist of alcoholic drinks (“beer”, “wine, “port, “whisky”, “vodka” and “rum”) or sodas (“Coke”, “Cassis”, “Sinas”, “Spa”, “tonic”, and “juice”). The attribute set consists of positive (“sociable”, “good”, “pleasant”, “nice”, “enjoyable” and “sympathetic”) and negative words (“antisocial”, “bad”, “unpleasant”, “stupid”, “obnoxious” and “tedious”). The Dutch words were matched for prevalence and number of syllables [63]. The stimulus words are presented on a 17 inch laptop (using Inquisit 3 desktop software).

The IAT has nine phases that come in one of two orders. Every phase consists of one practice block and either one or two measurement blocks. Each block consists of 48 randomly selected words. Attribute words (positive or negative) are presented in black (fontsize 14) in the middle of the screen. Feedback appears in red (fontsize 16) below the stimuli words. In case of a wrong response, the word “ERROR” appears on the screen. After responses that are too fast (<150 ms) or too slow (>3 s), feedback follows (“TOO FAST” or “TOO SLOW”) with a warning beep. The category word or words (alcohol or soda words) are presented at the top of the screen, on the left or the right side, depending on the required response (as in Greenwald et al., 1998). The interstimulus interval is 250 ms.

##### Self-efficacy list for drug users (SELD; [54])

The 19-item SELD measures situational abstinence self-efficacy. The authors state that self-efficacy should not be considered a stable quality of a person, but depends on the situation in which the person finds oneself. Three correlated dimensions were found: environmental factors, negative mood and positive mood. SELD scores correlated as expected with severity of drug use. Although it is a reliable and valid instrument to measure self-efficacy in substance users, test-retest reliability and predictive validity remains to be established.

#### Measurements – effects on quality of life

##### EuroQol-5D (EQ-5D; [55]

The EQ-5D is a short (14 VAS scales (0–100)) generic health-related quality of life (HRQOL) patient-report questionnaire which assesses 5 health dimensions (mobility, self-care, usual activities, pain/discomfort and anxiety/depression). Responses in each dimension are divided into three ordinal levels coded: no, moderate or extreme problems. Test-retest reliability and convergent validity are satisfactory in different populations [86,87] but the EQ-5D may show a moderate ceiling effect, thus indices seem less responsive for detecting meaningful clinical differences in alcohol-dependent patients compared to other instruments [88].

##### Community reinforcement approach happiness scale (CRA-HS; [48])

The CRA-HS is a multidimensional instrument that examines a wide variety of life areas on 10-point Likert scales. It forms the foundation of a CRA treatment plan and is used to determine individuals’ current “happiness” in terms of satisfaction or quality of life. It casts light on the severity of problems for each area and subsequently can be used to evaluate treatment outcome by repeatedly assessing changes that occur during treatment. The instrument encompasses life areas such as drinking (or illegal drug use), household, job/education, money management, social life, personal habits, romantic relation, family relationships, legal issues, emotional life, communication, health and general happiness. Preliminary results show that the instrument has overall satisfactory internal consistency and sensitivity to changes over a follow-up period of a month [56]. In addition, scores correlated with the level of rewarding activities.

#### Measurements – safety, acceptability and feasibility of treatment

All regular care providers, EMDR therapists involved in the study, treatment supervisors and physicians have been instructed to report adverse events (AE) and serious adverse events (SAE) which may arise during treatment. At T1 all regular therapists are e-mailed with questions about possible SAE that may have gone unreported between T0 and T1. In addition, all participants are questioned about possible AE and SAE at all time-points. Finally, participants in the EMDR + TAU group keep a daily diary during EMDR treatment in which they are instructed to report (S)AE.

At each time-point participants answer questions about opinions and expectations regarding the treatment (be it TAU or EMDR + TAU) they received, the credibility and burden of treatment. In addition, at the end (completed or not) of each EMDR treatment, the EMDR therapist is required to answer some exit-questions regarding the feasibility of the treatment for this particular participant. More generally, before the first and after the last EMDR treatment in the data collection phase EMDR therapists fill in a questionnaire about their opinions and expectations regarding the EMDR training and supervision, the credibility and burden of treatment.

#### Fidelity checks

EMDR therapists receive one full and two half-days of training in the EMDR treatment protocol. All EMDR therapists will be supervised by an EMDR consultant to guide optimal application of the treatment protocol. Monthly, two-hour group supervision supports the EMDR therapist for the whole duration of the data collection phase. Between supervision sessions, the consultant is available for consultation by email. All EMDR treatment sessions will be videotaped. A random selection will be rated for treatment fidelity by several independent raters with EMDRIA approved basic training. Deviations from the protocol will be reported to the consultant and EMDR therapist.

Since participants are randomized and TAU is therefore assumed equal in both groups, no fidelity checks are done regarding TAU.

### Analyses

All analyses will be executed using SPSS. Summary tables will be provided for all baseline, end of treatment and follow-up variables. Data will be summarised using frequency tables (with confidence intervals when applicable), and descriptive statistics (mean, median, interquartile range, number and percentages of participants and two-sided, 95% confidence intervals when appropriate).

Changes in the number of heavy drinking days in the past 30 days will be used to analyze the efficacy and effectiveness of EMDR. Since this primary outcome variable is continuous, a repeated measures ANOVA will be carried out with 2 group (EMDR + TAU versus TAU) and 4 time (pre-, post-treatment, 1 and 6 month follow-up) factors. Randomization stratum will be used as a covariate. Effect sizes will be calculated.

All allocated participants will be included in the primary analysis (intention-to-treat, ITT). Since an ideal ITT requires a complete set of data [89], multiple imputation (or a similar strategy) will be used for missing data, if possible. In addition a per-protocol (PP or completers) analysis will be done. ITT analyses will provide insight into the effectiveness of the treatment in real life whereas PP analysis gives insight into the efficacy or the ability for the treatment to reach its intended effect under ideal circumstances.

Repeated measures of continuous secondary variables such as time to first alcohol consumption, number of total drinks consumed and average drinks per occasion in the past 30 days, severity of the dependence, level of rumination, negative affect, desire thinking, craving, coping self-efficacy, quality of life, biomarkers will be analyzed by carrying out a repeated measures ANOVA with 2 group (EMDR + TAU versus TAU) and 3 (post treatment versus 1 versus 6 month follow-up: time to first drink variable derived from TLFB data) or 4 time (pre- versus post-treatment versus 1 versus 6 month follow-up: all other data) factors.

Distribution of premature treatment termination (PTT), a categorical variable, will be analysed using chi-square tests. A range of baseline clinical characteristics potentially associated with subsequent PTT will be examined. A logistic regression analysis will be conducted to examine the relative contribution of key variables to PTT status. A Kaplan–Meier survival curve will be used to illustrate the distribution of PTT prior to the completion threshold at session 5.

Group equivalence will be analyzed using independent t-tests to compare the experimental with the control group on continuous baseline or pretreatment measures of severity and duration of alcohol dependence, motivation to change, coping self-efficacy, quality of life, craving, desire thinking, rumination, negative affect and biomarker levels of GGT and CDT. Group equivalence of categorical variables, such as level of education, history of other substance use, specific contents of TAU use of anti-craving, abstinence enforcing or other psychoactive medication during the study treatment phase and co morbidity will be analyzed using chi-square tests.

Variables such as use of anti-craving, abstinence enforcing or other psychoactive medication, time-in-treatment, craving, rumination, negative affect and desire thinking at T0, may be used as covariates after determining correlations with other primary and secondary outcome measures.

## Discussion

This RCT aims to extend previous findings [15] by including a larger number of participants, using reaction time tasks and bloodsamples in addition to participant-report questionnaires and scales, excludes the principal investigator from treatment and data-collection and uses an outpatient instead of an inpatient setting. A strength of the study is that it is a multi-site trial, enhancing the internal and external validity of the interventions. Another strength is that the exclusion criteria are limited, enhancing the generalizability of findings. In addition, by identifying mediators, moderators and predictors of treatment outcome theoretical models of relapse may be expanded.

The main goal of the study is to determine whether EMDR, when added to TAU, reduces drinking behavior and relapse rates further than TAU only. Even a relative small effect size may be relevant, when achieved with a limited dose of EMDR. In addition, EMDR may provide an important adjunct to current treatment options for patients suffering from craving, negative affect and rumination. In both cases the quality of life is expected to improve.

Finally, the study is aimed at establishing whether the application of EMDR aimed at addiction is feasible and safe in this patient population, even when patients still use substances. Clinicians may be hesitant to use EMDR in this population because they fear triggering craving and subsequent relapse.

## Trial status

Currently recruiting participants and started with data collection.

## Abbreviations

ACRA: Adolescent Community Reinforcement Approach
AE: Adverse events
ANOVA: Analysis of variance
AUDIT: Alcohol use disorders identification test
CDT: Carbohydrate-deficient transferrin
CIDI: Composite international diagnostic interview
CRA: Community reinforcement approach
CRA-HS: Community reinforcement approach happiness scale
DSM-IV-TR: Diagnostic and statistical manual of mental disorders (4th ed., text revision)
DTQ: Desire thinking questionnaire
EMDR: Eye movement desensitization and reprocessing
EQ-5D™: EuroQol - 5 dimensions
FACT: Function assertive community therapy
GGT: serum γ-glutamyltransferase
IAT: Implicit association task
MATE: Measurements in the addictions for triage and evaluation
MDFT: Multidimensional family therapy
M.I.N.I.-plus: M.I.N.I. international neuropsychiatric interview-plus
PACS: Penn alcohol craving scale
PANAS: Positive and negative affect schedule
PANAS-SF: Positive and negative affect schedule – short form
PTQ: Perseverative thinking questionnaire
PTSD: Posttraumatic stress disorder
RA: Research assistant
RCQ-D: Readiness to change questionnaire - dutch version
RCT: Randomized controlled trial
SAE: Serious adverse events
SD: Standard deviation
SELD: Self-efficacy list for drug users
TAU: Treatment as usual
TLFB: Timeline followback
VAS: Visual analogue scale

## References

[1] Wisselink DJ, Kuijpers WJG, Mol A (2014). Kerncijfers verslavingszorg 2013.

[2] Sinha R (2011). New findings on biological factors predicting addiction relapse vulnerability. Curr Psychiatry Rep.

[3] Koob GF (2008). A role for brain stress systems in addiction. Neuron..

[4] Rosenberg H (2009). Clinical and laboratory assessment of the subjective experience of drug craving. Clin Psychol Rev..

[5] Oslin DW, Cary M, Slaymaker V, Colleran C, Blow FC (2009). Daily ratings measures of alcohol craving during an inpatient stay define subtypes of alcohol addiction that predict subsequent risk for resumption of drinking. Drug Alcohol Depend..

[6] Caselli G, Spada MM (2011). The desire thinking questionnaire: development and psychometric properties. Addict Behav.

[7] Caselli G, Ferla M, Mezzaluna C, Rovetto F, Spada MM (2012). Desire thinking across the continuum of drinking behavior. Eur Addict Res..

[8] Caselli G, Ferretti C, Leoni M, Rebecchi D, Rovetto F, Spada MM (2010). Rumination as a predictor of drinking behaviour in alcohol abusers: a prospective study. Addiction.

[9] Caselli G, Gemelli A, Querci S, Lugli AM, Canfora F, Annovi C (2013). The effect of rumination on craving across the continuum of drinking behaviour. Addict Behav..

[10] Thomsen DK, Jorgensen MM, Mehlsen MY, Zachariae R (2004). The influence of rumination and defensiveness on negative affect in response to experimental stress. Scand J Psychol..

[11] Cheetham A, Allen NB, Yucel M, Lubman DI (2010). The role of affective dysregulation in drug addiction. Clin Psychol Rev..

[12] Field M, Cox WM (2008). Attentional bias in addictive behaviors: a review of its development, causes, and consequences. Drug Alcohol Depend..

[13] Stacy AW, Wiers RW (2010). Implicit cognition and addiction: a tool for explaining paradoxical behavior. Annu Rev Clin Psychol..

[14] Adamson SJ, Sellman JD, Frampton CM (2009). Patient predictors of alcohol treatment outcome: a systematic review. J Subst Abus Treat..

[15] Hase M, Schallmayer S, Sack M (2008). EMDR reprocessing of the addiction memory: pretreatment, posttreatment, and 1-Month follow-up. J EMDR Pract Res.

[16] Shapiro F (2007). EMDR, adaptive information processing and case-conceptualization. J EMDR Pract Res.

[17] Gunter RW, Bodner GE (2008). How eye movements affect unpleasant memories: support for a working-memory account. Behav Res Ther.

[18] Hensley BJ (2012). Adaptive information processing, targeting, the standard protocol, and strategies for successful outcomes in EMDR reprocessing. J EMDR Pract Res.

[19] Engelhard IM, Van Uijen SL, Van den Hout MA (2010). The impact of taxing working memory on negative and positive memories. Eur J Psychotraumatol..

[20] Logie R, De Jongh A (2014). The “Flashforward procedure”: confronting the catastrophe. J EMDR Pract Res.

[21] Bisson JI, Ehlers A, Matthews R, Pilling S, Richards D, Turner S (2007). Psychological treatments for chronic post-traumatic stress disorder: systematic review and meta-analysis. Br J Psychiatry.

[22] Leeds AM (2009). A guide to the standard EMDR protocols for clinicians, supervisors, and consultants.

[23] Abel NJ, O’Brien JM (2010). EMDR treatment of comorbid PTSD and alcohol dependence: a case example. J EMDR Pract Res.

[24] Cecero JJ, Carroll KM (2000). Using eye movement desensitisation and reprocessing to reduce cocaine cravings. Am J Psychiatry.

[25] Hornsveld H, Hornsveld H, Berendsen S (2009). Casus 25: nog eentje – daarna stop ik. Een mislukte stoppen-met-rokenbehandeling. Casusboek EMDR. 25 voorbeelden uit de praktijk.

[26] Marich J (2009). EMDR in the addiction continuing care process. J EMDR Pract Res.

[27] Marich J (2010). Eye movement desensitization and reprocessing in addiction continuing care: a phenomenological study of women in recovery. Psychol Addict Behav.

[28] Shapiro F, Vogelmann-Sine S, Sine LF (1994). Eye movement desensitization and reprocessing: treating trauma and substance abuse. J Psychoactive Drugs.

[29] Tsoutsa A, Fotopoulos D, Zakynthinos S, Katsaounou P (2014). Treatment of tobacco addiction using the Feeling-State Addiction Protocol (FSAP) of the Eye Movement Desensitization and Reprocessing (EMDR) treatment. Tob Induc Dis.

[30] Van Uitert-Levy T (2010). Is EMDR een alternatief voor de behandeling van trek in verslavende middelen?. Verslaving..

[31] Rooijmans J, Rosenkamp NHG, Vernholt P, Visscher RA (2012). The effect of eye movements on craving, pleasantness and vividness in smokers. Soc Cosmos..

[32] Bae H, Han C, Kim D (2013). Desensitization of triggers and urge reprocessing for pathological gambling: a case series. J Gambl Stud [online]..

[33] Bae H, Kim D (2012). Desensitization of triggers and urge reprocessing for an adolescent with internet addiction disorder. J EMDR Pract Res.

[34] Cox RP, Howard MD (2007). Utilization of EMDR in the treatment of sexual addiction: a case study. Sex Addict Compulsivity.

[35] Henry SL (1996). Pathological gambling: etiological considerations and treatment efficacy of eye movement desensitization/reprocessing. J Gambl Stud..

[36] Miller R (2010). Feeling-state theory and the impulse-control disorder protocol. Traumatology.

[37] Miller R (2012). Treatment of behavioral addictions utilizing the feeling-state addiction protocol: a multiple baseline study. J EMDR Pract Res.

[38] Hase M, Crav E, Luber M (2009). An EMDR approach to treat substance abuse and addiction. Eye movement desensitization (EMDR) scripted protocols: special populations.

[39] Popky AJ, Shapiro R (2005). DeTUR, an urge reduction protocol for addictions and dysfunctional behaviors. EMDR solutions: pathways to healing.

[40] Laudet AB (2011). The case for considering quality of life in addiction research and clinical practice. Addict Sci Clin Pract.

[41] American psychiatric association: *diagnostic and statistical manual of mental disorders* (4th ed., text revision). Washington, DC: Author; 2000.

[42] Boter H, Van Delden JJM, De Haan RJ, Rinkel GJE (2003). Modified informed consent procedure: consent to postponed information. BMJ..

[43] Sheehan DV, Lecrubier Y, Harnett-Sheehan K, Amorim P, Janavs J, Weiller E (1998). The MINI International Neuropsychiatric Interview (M.I.N.I.): the development and validation of a structured diagnostic psychiatric interview. J Clin Psychiatry.

[44] Schippers GM, Broekman TG: *MATE, Measurements in the Addictions for Triage and Evaluation. Development of an instrument assessing patient characteristics in substance abuse treatment. Final report ZonMw/Resultaten Scoren-project nr 31000068*. Amsterdam, AIAR, AMC-Amsterdam & Bureau Bêta, Nijmegen; 2007. [http://www.mateinfo.eu/pubs/31000068.pdf]

[45] Random allocation software [http://mahmoodsaghaei.tripod.com/Softwares/randalloc.html]

[46] Merkx MJM, Schippers GM, Koeter MWJ, Vuijk PJ, Poch M, Kronemeijer H (2013). Predictive validity of treatment allocation guidelines on drinking outcome in alcohol-dependent patients. Addict Behav..

[47] Faul F, Erdfelder E, Lang A-G, Buchner A (2007). G*Power 3: a flexible statistical power analysis program for the social, behavioral, and biomedical sciences. Behav Res Methods..

[48] Meyers RJ, Smith JE (1995). Clinical guide to alcohol treatment: the community reinforcement approach.

[49] Rowe CL (2010). Multidimensional family therapy: addressing co-occurring substance abuse and other problems among adolescents with comprehensive family-based treatment. Child Adolesc Psychiatr Clin N Am.

[50] Drukker M, Van Os J, Sytema S, Driessen G, Visser E, Delespaul P (2011). Function assertive community treatment (FACT) and psychiatric service use in patients diagnosed with severe mental illness. Epidemiol Psychiatr Sci..

[51] Knipe J, Luber M (2009). Dysfunctional positive affect: codependence or obsession with self-defeating behavior. Eye movement desensitization (EMDR) scripted protocols: special populations.

[52] Rollnick S, Heather N, Gold R, Hall W (1992). Development of a short ‘readiness to change’ questionnaire for use in brief, opportunistic interventions among excessive drinkers. Br J Addict..

[53] Saunders JB, Aasland OG, Babor TF, DeLaFuente JR, Grant M (1993). Development of the Alcohol Use Disorders Identification Test (AUDIT): WHO collaborative project on early detection of persons with harmful alcohol consumption II. Addiction..

[54] De Weert-Van Oene GH, Breteler MH, Schippers GM, Schrijvers AJ (2000). The validity of the Self-Efficacy List for Drug Users (SELD). Addict Behav.

[55] Rabin R, De Charro F (2001). EQ-5D: a measure of health status from the EuroQol Group. Ann Med.

[56] Dijkstra BAG, Roozen HG (2012). Patients’ improvements measured with the pleasant activities list and the community reinforcement approach happiness scale: preliminary results. Addict Disord Their Treat.

[57] Flannery BA, Volpicelli RJ, Pettinati HM (1999). Psychometric properties of the Penn Alcohol Craving Scale. Alcohol Clin Exp Res.

[58] Thompson ER (2007). Development and validation of an internationally reliable short-form of the Positive And Negative Affect Schedule (PANAS). J Cross Cult Psychol.

[59] Ehring TWA, Zetsche U, Weidacker K, Wahl K, Schönfeld S, Ehlers A (2011). The Perseverative Thinking Questionnaire (PTQ): validation of a content-independent measure of repetitive negative thinking. J Behav Ther Exp Psychiatry.

[60] Perseus web survey software [http://www.perseusuk.co.uk/index.html]

[61] Sobell LC, Sobell MB, Litten RZ, Allen J (1992). Timeline followback: a technique for assessing self-reported alcohol consumption. Measuring alcohol consumption: psychosocial and biological methods.

[62] Williams JM, Mathews A, MacLeod C (1996). The emotional Stroop task and psychopathology. Psychol Bull.

[63] Wiers RW, Van Woerden N, Smulders FT, De Jong PJ (2002). Implicit and explicit alcohol-related cognitions in heavy and light drinkers. J Abnorm Psychol..

[64] Inquisit 3 desktop version software [http://www.millisecond.com/]

[65] Niemelä O (2007). Biomarkers in alcoholism. Clin Chim Acta..

[66] World Health Organization (1997). Composite International Diagnostic Interview (CIDI) (Version 2.1).

[67] Van Vliet I, De Beurs E (2007). The Mini-International Neuropsychiatric Interview. A brief structured diagnostic psychiatric interview for DSM-IV and ICD-10 psychiatric disorders. Tijdschr Psychiatr.

[68] Lecrubier Y, Sheehan DV, Weiller E, Amorim P, Bonora I, Harnett Sheehan K (1997). The MINI International Neuropsychiatric Interview (M.I.N.I.), a short diagnostic structured interview: reliability and validity according to the CIDI. Eur Psychiatry.

[69] Schippers GM. Broekman TG: (2010). ^*^ Corresponding authorBabor T, Higgins-Biddle JC, Saunders J, & Monteiro MG (2001). [http://www.mateinfo.nl/audit/audit-nl.pdf]

[70] Dolman JM, Hawkes ND (2005). Combining the audit questionnaire and biochemical markers to assess alcohol use and risk of alcohol withdrawal in medical inpatients. Alcohol.

[71] Gache P, Michaud P, Landry U, Accietto C, Arfaoui S, Wenger O (2005). The Alcohol Use Disorders Identification Test (AUDIT) as a screening tool for excessive drinking in primary care: reliability and validity of a French version. Alcohol Clin Exp Res..

[72] Trimbosinstituut/Landelijke Stuurgroep Multidisciplinaire Richtlijnontwikkeling in de GGZ (2009). Multidisciplinaire Richtlijn Stoornissen in het gebruik van alcohol. Richtlijn voor de diagnostiek en behandeling van volwassen patiënten met een stoornis in het gebruik van alcohol.

[73] De Fuentes-Merillas L, De Jong CAJ, Schippers GM (2002). Reliability and validity of the Dutch version of the Readiness to Change Questionnaire. Alcohol Alcohol.

[74] Prochaska J, DiClemente C (1983). Stages and processes of self-change of smoking: toward an integrative model of change. J Consult Clin Psychol.

[75] Heather N, Rollnick S, Bell A (1993). Predictive validity of the Readiness to Change Questionnaire. Addiction.

[76] Addington J, El-Guebaly N, Duchak V, Hodgins D (1999). Using measures of readiness to change in individuals with schizophrenia. J Drug Alcohol Abuse..

[77] West R (2005). Time for a change: putting the transtheoretical (stages of change) model to rest. Addiction..

[78] Flannery BA, Poole SA, Gallop RJ, Volpincelli JR (2003). Alcohol craving predicts drinking during treatment: an analysis of three assessment instruments. J Stud Alcohol.

[79] Kavanagh DJ, Andrade J, May J (2005). Imaginary relish and exquisite torture: the elaborated intrusion theory of desire. Psychol Rev.

[80] Boon MTG, Peeters FPML (1999). Affectieve dimensies bij depressie en angst. Tijdschr Psychiatr.

[81] Watson D, Clark LA, Tellegen A (1988). Development and validation of brief measures of positive and negative affect: the PANAS scales. J Pers Soc Psychol..

[82] Ehring TWA, Raes F, Weidacker K, Emmelkamp PMG (2012). Validation of the Perseverative Thinking Questionnaire – Dutch version (PTQ-NL). Eur J Psychol Assess..

[83] Stroop JR (1935). Studies of interference in serial verbal reaction. J Exp Psychol..

[84] Greenwald AG, Farnham SD (2000). Using the Implicit Association Test to measure self-esteem and self-concept. J Pers Soc Psychol..

[85] Alcohol IAT from the Inquisit task library [http://www.millisecond.com/download/library/IAT/AlcoholIAT/]

[86] Salén BA, Spangfort EV, Nygren AL, Nordemar R (1994). The Disability Rating Index: an instrument for the assessment of disability in clinical settings. J Clin Epidemiol.

[87] Dyer M, Goldsmith KA (2010). A review of health utilities using the EQ-5D in studies of cardiovascular disease. Health Qual Life Outcomes..

[88] Günther OH, Roick C, Angermeyer MC, Konig HH (2008). Responsiveness of EQ-5D utility indices in alcohol-dependent patients. Drug Alcohol Depend.

[89] Armijo-Olivo S, Warren S, Magee D (2009). Intention to treat analysis, compliance, drop-outs and how to deal with missing data in clinical research: a review. Phys Ther Rev..

